# Human-annotated rationales and explainable text classification: a survey

**DOI:** 10.3389/frai.2024.1260952

**Published:** 2024-05-24

**Authors:** Elize Herrewijnen, Dong Nguyen, Floris Bex, Kees van Deemter

**Affiliations:** ^1^Department of Information & Computing Sciences, Utrecht University, Utrecht, Netherlands; ^2^National Police Lab AI, Netherlands Police, Driebergen, Netherlands; ^3^Tilburg Institute for Law, Technology and Society, Tilburg University, Tilburg, Netherlands

**Keywords:** annotator rationales, natural language explanations, explainable artificial intelligence, data collection, machine learning, rationale agreement, text classification, human-annotated rationales

## Abstract

Asking annotators to explain “why” they labeled an instance yields annotator rationales: natural language explanations that provide reasons for classifications. In this work, we survey the collection and use of annotator rationales. Human-annotated rationales can improve data quality and form a valuable resource for improving machine learning models. Moreover, human-annotated rationales can inspire the construction and evaluation of model-annotated rationales, which can play an important role in explainable artificial intelligence.

## 1 Introduction

With an ever-growing number of applications and users, it is important that language-based artificial intelligence (AI) models can be explained in a human-like way. Natural language explanations that are provided by humans, often referred to as “annotator rationales”, are a promising resource for building explainable AI (XAI) systems.

Seminal works (Zaidan et al., 2007, 2008; Zaidan and Eisner, 2008) have collected annotator rationales by asking human annotators to highlight parts of a text to justify “why” that text should receive a certain label. The term “annotator rationale” has since been used with different meanings; for example, as human-annotated highlights in a text (Volkova and Yarowsky, 2014; Kutlu et al., 2020), as human-annotated free-text comments (Kartal and Kutlu, 2020), or as highlights generated by a machine learning (ML) model (Yessenalina et al., 2010). In this work, we consider *annotator rationales* to be natural language explanations (i.e., rationales) produced by the annotator (e.g., a human or an ML model).

While annotator rationales have been collected and used within the field of natural language processing (NLP), to our knowledge, no overview to guide those interested in using annotator rationales in NLP exists. Therefore, this article aims to provide insight into the lessons learned when it comes to collecting and using annotator rationales in NLP. We do this by surveying the use of annotator rationales in the field of NLP, specifically for explainable text classification.

### 1.1 Scope and selection criteria

Rationales have been used in many NLP tasks, e.g., natural language inference (Camburu et al., 2018; Kumar and Talukdar, 2020), next word prediction (Vafa et al., 2021), question answering (Lamm et al., 2021), and translation quality (Fomicheva et al., 2021). In this survey, we mainly focus on explainable single-input text classification, thereby limiting explanation to identifying or describing relevant parts of a text instance. Multi-input tasks bring additional challenges when generating rationales; for example, it is unclear whether the explanations should refer to all inputs (e.g., the question and the answer in a question-answering task), or a selection of inputs (e.g., only the answer in a question-answering task).

Using Google Scholar, we select relevant literature for our survey by looking into related work that cites (Zaidan et al., 2007) and work that uses the terms “annotator rationales”, “rationales” and “natural language explanations”. Moreover, we apply the following criteria to select relevant literature:

- The work relates to (explainable) NLP.
- The work collects and/or uses natural language explanations that are provided by humans.
- The work involves a single-input text classification task.

We occasionally include studies outside these criteria, when they give relevant insight into annotator rationales. We survey literature published up to 2023.

### 1.2 Related surveys

Natural language explanations for AI have been surveyed in related work: there are, for example, surveys on explainable NLP (Danilevsky et al., 2020), general XAI methods that generate natural language explanations (Cambria et al., 2023; Gurrapu et al., 2023), datasets for explainable NLP (Wiegreffe and Marasovic, 2021), and using human rationales for improving ML models (Hartmann and Sonntag, 2022). Complementary to the above surveys, this work focuses specifically on annotator rationales as provided by *humans* and their application in *explainable* text classification.

### 1.3 Outline

This article is structured as follows: first, we propose a conceptual framework for rationales in Section 2. Then, we discuss how annotator rationales have been collected from human annotators for various tasks, and through various annotation schemes (Section 3). We provide an overview of datasets containing annotator rationales and invite the community to collaboratively update this overview using GitHub.^1^ We then proceed to survey how human annotator rationales are used in explainable text classification. We first provide a brief introduction to basic concepts of XAI (Section 4), and then discuss how human annotator rationales are used for generating and evaluating rationales in explainable text classification (Section 5). We conclude our survey with a list of concrete suggestions for the use of rationales in XAI (Section 6).

## 2 A framework for rationales

**Figure 1:**
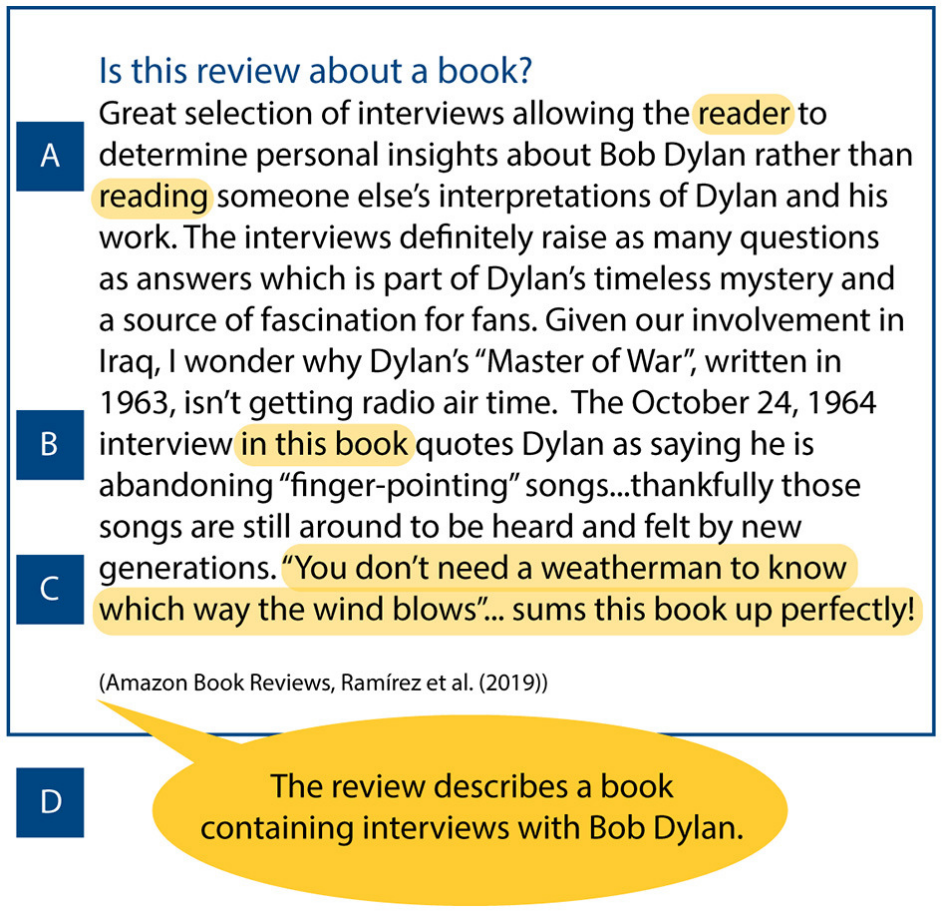
A book review from the Amazon Book Reviews dataset (Raḿırez et al., 2019). The task is assigning the label aboutBook to reviews that are written about books. For this instance, the annotator has labeled the review with the aboutBook label. The highlighted parts of the text are examples of extractive word (A), snippet (B), and sentence (C) rationales. The speech bubble (D) shows an abstractive sentence rationale. These rationales explain why the review is annotated with the aboutBook label.

In the following section, we propose a conceptual framework for *rationales*: explanations in natural language format (Ehsan et al., 2019). Figure 1 provides examples of rationales. As humans often explain their decisions through natural language, it is reasonable to assume that this type of explanation is suitable for explaining AI to non-technical users (Miller et al., 2017; Cambria et al., 2023; Mukhtar et al., 2023). Furthermore, rationales can adopt domain-specific jargon, tailoring the explanation to domain expert knowledge (e.g., medical practice Meldo et al., 2020). We describe various kinds of rationales in the following paragraphs.

### 2.1 Form

#### 2.1.1 Granularity

Rationales appear in various granularities (see Figure 1). The highest granularity is collections of *words* extracted from a text, or keywords describing an instance. *Snippet*-level rationales consist of multiple consecutive words, and *sentence*-level rationales consist of single sentences. Finally, *paragraph*-level rationales are multi-sentence natural language explanations. Note that a rationale can consist of multiple text spans (e.g., snippets), that together make up one rationale explaining a decision. Some granularities may be more suitable than others; for example, Jain et al. (2020) find that humans prefer snippets to words. However, few studies examine the suitability of different rationale granularities.

#### 2.1.2 Extractive and abstractive

We distinguish between two types of rationales; the first is *extractive* rationales, such as words, sentences, and snippets which are parts of the input text. Extractive rationales are also referred to as excerpts (McDonnell et al., 2016) or highlights (Zaidan et al., 2007).

The second type of rationale is an *abstractive* rationale. These are free-text natural language explanations that *refer* to the input text, but are not (exact) parts of the input text. We adopt the term abstractive from automated text summarization (Lin and Ng, 2019; Gurrapu et al., 2023). Abstractive rationales are more difficult to evaluate and may increase annotation time (Kutlu et al., 2020; Wiegreffe et al., 2021), but they allow annotators to intuitively provide explanations using an unrestricted vocabulary.

#### 2.1.3 Categorical and numerical

Most rationales annotated by humans are *categorical*. Human annotators often select specific spans of text that explain their decision, resulting in binary rationales. However, annotations can also occur on a more nuanced level, where spans of text can explain or contradict a decision (Kutlu et al., 2020; Sullivan et al., 2022). Some rationales, in particular extractive rationales, are composed of elements that are assigned *numeric* values. For example, when the word “great” has a value of 0.9 and “okay” has a value of 0.5, this could suggest that “great” is more relevant than “okay”. Numerical rationales can be collected or constructed by, for example, combining rationale values across annotators (e.g., 70% agrees “great” is a rationale) (Mathew et al., 2021), asking new annotators to rate rationales based on usefulness (Raḿırez et al., 2019), or using machine learning techniques to assign weights to text spans [e.g., the attention mechanism (Bao et al., 2018)].

### 2.2 Exhaustiveness

Rationales vary in degrees of exhaustiveness. Take for example the annotation instructions by Sen et al. (2020): “*highlight (ALL) the words that are indicative of the chosen sentiment”*; here, the goal is to annotate an *exhaustive* rationale, i.e., *all* text spans (e.g., words, snippets, sentences) that explain a decision (DeYoung et al., 2020; Sen et al., 2021). Alternatively, take the annotation instructions by Abedin et al. (2011): “*annotators are asked to ‘do their best to mark enough rationales to provide convincing support for the class of interest', but are not expected to ‘go out of their way to mark everything.”'*; here, the rationale is probably not exhaustive, but is nevertheless sufficient to explain the decision.

### 2.3 Human-annotated rationales and model-annotated rationales

We identify two categories of annotator rationales according to the type of annotator. *Human-annotated* rationales (hARs) are provided by human annotators—e.g., students, domain experts, or crowd workers—and *model-annotated* rationales (mARs) are provided by an ML model.

## 3 Human-annotated rationales

Next up, we discuss human-annotated rationales (hARs). Specifically, we outline several aims and benefits of collecting hARs (Section 3.1), and the lessons learned from annotation setups collecting hARs (Section 3.2). Table 1 provides an overview of datasets containing hARs.^2^

**Table 1:** Overview of datasets with human-annotated rationales in the literature.

						**Collection aim**						
**Related work**	**Classification task**	**Granularity**	**Form**		**Value type**	**Improving ML**	**Task insight**	**Data quality**	**Gold explanation**	**Data generation**	**Annotator**	**Name (if available)**
Zaidan et al. (2007)	Sentiment	Sn	E		C	✓					O	IMDB
Titov and McDonald (2008)	Sentiment	Se	E		C				✓		O	TripAdvisor^*^
Yano et al. (2010)	Bias	Sn	E		C		✓				Cw	
Abedin et al. (2011)	Aviation incident causes	Sn	E		C	✓				✓	O	ASRS
McAuley et al. (2012)	Sentiment	S	E		C	✓			✓		De	BeerAdvocate
Saleem et al. (2012)	Medical	Sn	E		C	✓					De	
Xia and Yetisgen-Yildiz (2012)	Medical	S		A	C			✓			De	
Tepper et al. (2013)	Medical	Sn	E		C	✓					De	CPIS/PNA
Marshall et al. (2015)	Bias	Sn	E		C				✓		De	RoB
McDonnell et al. (2016)	Webpage relevance	Se	E	A	C			✓			Cw	
Bao et al. (2018)	Sentiment	Sn	E		C	✓					O	BeerAdvocate^*^
Carton et al. (2018)	Personal attacks	Sn	E		C				✓		O	
Chhatwal et al. (2018)	Legal	Sn	E	A	C	✓					De	
Kaushik et al. (2019)	Sentiment	Sn	E		C	✓					Cw	IMDB^*^
Raḿırez et al. (2019)	Topic	Sn	E	A	N		✓				Cw	SLR
Raḿırez et al. (2019)	Topic	Sn	E	A	C		✓				Cw	Amazon
Wang et al. (2020)	Sentiment	Se		A	C					✓	Cw	SemEval-2014^*^
Hasanain et al. (2020)	Topic	Se	E	A	C	✓	✓		✓		De	ArTest
Kanchinadam et al. (2020)	Sentiment	Sn	E		C	✓					Cw	IMDB^*^
Kartal and Kutlu (2020)	Check-worthy claims	Sn		A	C		✓				O	TrClaim-19
Kreiss et al. (2020)	Guilt	Sn	E		C	✓	✓				Cw	SuspectGuilt
Kutlu et al. (2020)	Webpage relevance	Se	E	A	C			✓			Cw	
Sap et al. (2020)	Abusive content	Se		A	C	✓			✓		Cw	SBIC
Sen et al. (2020)	Sentiment	Sn	E		C				✓		Cw	Yelp-HAT
Arous et al. (2021)	Topic	Sn	E		C	✓			✓		Cw	Wiki-Tech
Chalkidis et al. (2021)	Legal	P	E		C				✓		De	ECtHR
Hayati et al. (2021)	Style	W	E		C				✓		Cw	Hummingbird
Jayaram and Allaway (2021)	Stance detection	W	E		C	✓					Cw	VAST^*^
Mohseni et al. (2021)	Sentiment	Sn	E		C				✓		Cw	IMDB^*^
Mohseni et al. (2021)	Topic	Sn	E		C				✓		Cw	20News^*^
Mathew et al. (2021)	Hate speech	Sn	E		N	✓			✓		Cw	HateXplain
Malik et al. (2021)	Legal	Se	E		C				✓		De	ILDC
Sharma et al. (2020)	Empathy expression	Sn	E		C				✓		Cw	EMH
Vidgen et al. (2021)	Abusive content	Sn	E		C				✓		De	CAD
El Zini et al. (2022)	Sentiment	W	E		C				✓		O	RottenTomatoes^*^
Chiang and Lee (2022)	Sentiment	Sn	E		C				✓		Cw	IMDB^*^
Guzman et al. (2022)	Forced labor indicators	Sn	E		C	✓					De	RaFoLa
Jørgensen et al. (2022)	Sentiment	W	E		C				✓		O	SST^*^
Lu et al. (2022)	Sentiment	Sn	E		C	✓					Cw	IMDB^*^
Sullivan et al. (2022)	Sentiment	Sn	E		C		✓				Cw	IMDB^*^
Wang et al. (2022)	Topic	Sn	E		C	✓					O	AIvsCR
Jakobsen et al. (2023)	Sentiment	W	E		C	✓					Cw	DynaSent^*^
Jakobsen et al. (2023)	Sentiment	W	E		C	✓					Cw	SST^*^

Granularity is abbreviated as Paragraphs, Sentences, Snippets, and Words. Form is abbreviated as Extractive and Abstractive. Values types are abbreviated as Categorical and Numerical. The annotator type is abbreviated as Crowd worker, Domain expert, and Other. When available, the name of the dataset is provided. The ^*^ symbol is used when human-annotated rationales are added to an already existing dataset.

### 3.1 Collection aims and benefits

After their introduction by Zaidan et al. (2007), human-annotated rationales have been collected for common tasks like sentiment and topic classification, but also for domain-specific tasks such as legal document classification (see Table 1). Furthermore, hARs have been collected with various aims, which we will discuss in the following section.

#### 3.1.1 Improving ML model performance

First, enriching datasets with hARs can be beneficial to ML model training; using the rationales, the ML model can be guided toward the most useful parts of the input for solving the task. Following Zaidan et al. (2007), many authors use hARs to improve their ML model performance (e.g., Saleem et al., 2012; Tepper et al., 2013; Krening et al., 2016; Chhatwal et al., 2018; Arous et al., 2021; Pruthi et al., 2022). In addition, the required amount of labeled training data can be substantially reduced (e.g., requiring only 10% of the original training data) by using hARs as enriched inputs (Arora and Nyberg, 2009; Sharma et al., 2015; Wang et al., 2022). See Hartmann and Sonntag (2022) for a survey on improving ML model (task) performance with human explanations.

Furthermore, hARs can teach ML models “valid reasons” for a classification, reducing spurious ML model behavior (Mathew et al., 2021; Chen et al., 2022; Joshi et al., 2022) and improving out-of-domain (OOD) performance (Lu et al., 2022).

#### 3.1.2 Task insight

Second, collecting hARs can help gain insight into the annotation task (Yano et al., 2010; Kartal and Kutlu, 2020). For example, Malik et al. (2021) identify annotator groups based on rationales in their legal document classification task: annotators used either “holistic reasoning” or “bare-minimum reasoning”. Another example is Kartal and Kutlu (2020), who identify important topics for their tweet classification task using hARs. Furthermore, there is an increasing awareness in NLP that disagreement in labeling is often informative and can point to differences in interpretation (Uma et al., 2022). Rationales can provide further insight into reasons for labeling disagreement, like annotator bias or instruction ambiguity (Kartal and Kutlu, 2020), especially when the labeling task is subjective (Sen et al., 2021).

#### 3.1.3 Data quality

Third, requesting annotator rationales from human annotators can improve data quality; forming a rationale requires annotators to consider their annotation more deeply, and collecting hARs thus reduces the number of classification mistakes made by human annotators (Kutlu et al., 2020). Moreover, hARs allow for effective data validation, for example through label aggregation [e.g., discarding labels with abnormal rationales (Sen et al., 2020)] or annotator discussion [e.g., providing rationales as arguments that annotators can respond to (Xia and Yetisgen-Yildiz, 2012; Drapeau et al., 2016; McDonnell et al., 2016; Kutlu et al., 2020)].

#### 3.1.4 Data generation

Fourth, hARs can be a valuable resource for data generation; for example, new data points can be created by removing extractive rationales from input texts (Zaidan et al., 2007), retaining only rationales in an input text (Abedin et al., 2011), or combining rationales from multiple input texts into a new instance (Volkova and Yarowsky, 2014). Furthermore, labeling functions can be constructed from both abstractive and extractive hARs (Li et al., 2015; Hancock et al., 2018). All things considered, hARs can be seen as rich labels: Hancock et al. (2018) even claim that for their experiment, “one explanation can be worth 100 labels”, and Sharma and Bilgic (2018) suggest that a document with rationales can be worth as many as 20 documents without rationales.

#### 3.1.5 Gold rationales

Fifth, human-annotated rationales are often collected for use as “gold rationales” (Table 1) to determine the quality of generated ML model explanations. In Section 5.2 we discuss this topic in more detail.

### 3.2 Insights from human rationale collection

In the following section, we discuss some insights from human rationale collection.

#### 3.2.1 Choice of annotators

As shown in Table 1, hARs are often collected with the aim of ML model improvement and as gold explanations. How beneficial hARs are to the various collection aims may depend on the annotator type; crowdsourcing platforms, like Mechanical Turk (Crowston, 2012), give access to a large group of annotators but allow for little data quality control. Here, requesting hARs from crowd workers may improve data quality by, for example, reducing the chance of annotators “cheating” (e.g., always selecting the second answer) (Kutlu et al., 2020). Apart from that, we expect that domain experts, who possess specific (domain) knowledge, can produce hARs that are highly useful for gaining task insight, generating data, or as gold explanations.

#### 3.2.2 Annotation instructions

Very little work explicitly instructs annotators to provide exhaustive rationales, e.g., “*highlight ALL words that reflect this sentiment”* (Sen et al., 2020) or “*we encouraged annotators to try their best to mark as many rationales as possible”* (Lu et al., 2022). In most cases, annotator rationales are collected using instructions like “*highlight rationales that are short and coherent, yet sufficient for supporting the label”* (Bao et al., 2018), “*select one or more sentences most useful for your decision”* (Raḿırez et al., 2019), “*why do you think so?”* (Hancock et al., 2018), or “*select the k most important words in the argument”* (Jayaram and Allaway, 2021). While it is often not stated explicitly, it is unlikely that collecting rationales using these instructions results in exhaustive rationales. This lack of clarity in instructions may pose issues when using hARs as gold rationales, which we will further discuss in Section 5.2.

#### 3.2.3 Effect on annotation time

One concern when asking annotators to provide hARs in annotation tasks is an increased annotation cost. Multiple studies report that the annotation time at most doubles when requesting extractive hARs for tasks like sentiment or topic classification (Hancock et al., 2018; Arous et al., 2021; Sullivan et al., 2022). This additional annotation time can be reduced when annotators gain experience in annotating rationales (McDonnell et al., 2016; Kutlu et al., 2020). A tentative conclusion is that annotators already subconsciously form rationales when performing the classification task, thus only requiring additional time to write or mark down the rationale (Zaidan et al., 2007; Kutlu et al., 2020). Whether this applies to abstractive hARs and more complex tasks, and how annotator experience is affected (i.e. task difficulty and enjoyment) is an open question. Alternatively to actively annotating rationales, techniques like eye-tracking might allow for passive rationale collection (Eberle et al., 2022); for example, construct a heatmap of relevant text snippets based on the annotator's gaze while performing a task.

#### 3.2.4 Inter-annotator agreement of rationales

Rationales are much more versatile than labels. Chiang and Lee (2022) and Sullivan et al. (2022) show that rationale annotation instructions greatly affect the form and exhaustiveness of resulting human-annotated rationales. When two rationales differ in form or size, it is difficult to calculate their inter-annotator agreement (Dumitrache et al., 2018; Kreiss et al., 2020; Malik et al., 2021; Guzman et al., 2022). Many studies report inter-annotator agreement on rationales using pairwise agreement (Zaidan et al., 2007; McDonnell et al., 2016; Wang et al., 2022) or Intersection-over-Union (also known as the Jaccard index) (Malik et al., 2021; Mathew et al., 2021; Guzman et al., 2022). Furthermore, some possible agreement measures are the Ratcliff-Obershelp metric (McDonnell et al., 2016), Cohen's Kappa (DeYoung et al., 2020), Krippendorff's alpha (Carton et al., 2018), ROUGE (Malik et al., 2021), and worker quality score (WQS) (Jayaram and Allaway, 2021). The above metrics often indicate that inter-annotator agreement for rationales is low, and varies between annotators and tasks (Carton et al., 2018; Malik et al., 2021; Wang et al., 2022). Nevertheless, inter-annotator agreement for rationales usually outperforms random baselines (Kreiss et al., 2020; Mathew et al., 2021). Overall, we expect that rationales collected from multiple annotators can capture useful information about the annotation task, and may be a versatile resource for developing (robust) ML models.

## 4 Explainable text classification

We now connect rationales to explainable AI (XAI), starting by outlining relevant XAI concepts. XAI revolves around explaining AI models, especially the ones based on machine learning (ML). An explanation can *globally* explain a complete ML model (i.e., elucidating the working of the model as a whole), or *locally* explain a specific input-output instance (e.g., highlighting relevant words in a text). Furthermore, an XAI method can be *model-agnostic*, meaning that it is applicable to any ML model, or *model-dependent*, meaning that it is applicable to a specific (group of) model(s).

### 4.1 Roles in XAI

In the XAI process, there are various actor roles that can be fulfilled by humans or ML models:

- The *annotator* maps inputs to outputs, also referred to as labeler, classifier, or decision-maker. Examples are human annotators and ML classification models. In this paper, we use the term “mapping” to refer to the annotator's internal decision-making process that maps inputs to outputs.
- The *explainer* explains the output produced by the annotator to the explanation receiver. For example, humans can justify their decision, or a surrogate explanation model like LIME (Ribeiro et al., 2016) can construe an ML model's behavior.
- The *explainee* receives the explainer's explanation. Explanations can be addressed to human users, but also to an ML model that learns a task using the explanation. Human users of AI systems can be broadly divided into three groups: *ML developers* have technical knowledge about the system; *domain experts* have domain-specific knowledge; *lay users* lack both technical and domain knowledge (Ribera and Lapedriza, 2019).
- The *validator* determines the quality of the explanation produced by the explainer. The desired qualities of an explanation depend on the explainee and the goal of the explanation.

In some cases, an actor can play multiple roles; for example, annotators providing a rationale are also explainers, and a human reviewing an explanation is both the explainee and the validator.

### 4.2 Explanation timing

**Figure 2:**
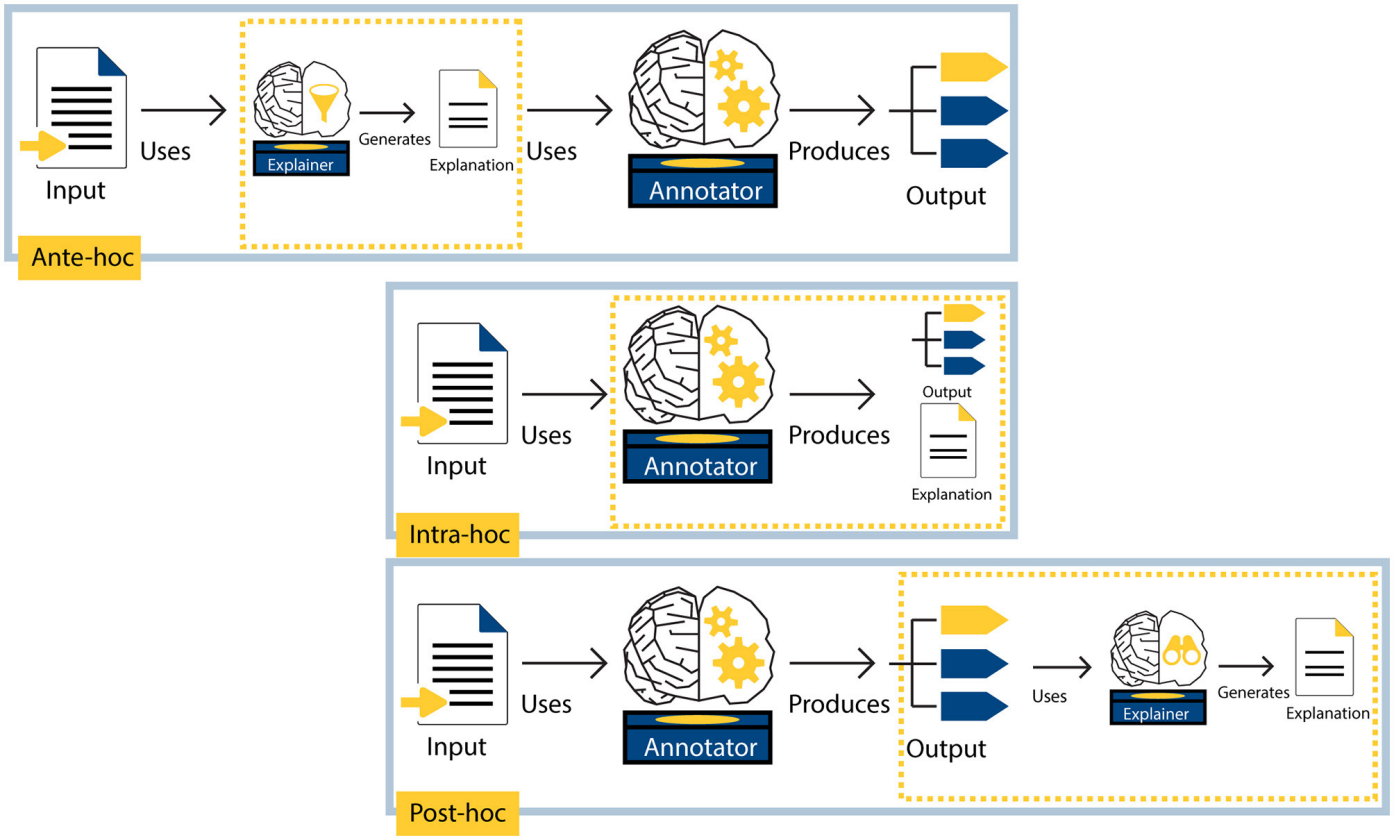
Three different timings in which explanations for AI systems can be generated. The **top row** shows an ante-hoc explanation setup, where the explainer model generates an explanation that is used as input by the annotator model to produce an output (e.g., Jain et al., 2020). The **middle row** shows an intra-hoc explanation setup, where the annotator model produces both an output and an explanation (e.g., Wiegreffe and Marasovic, 2021). The **bottom row** shows a *post-hoc* explainer that uses the output produced by the annotator model to generate an explanation (e.g., Ribeiro et al., 2016).

Zaidan et al. (2007) asked human annotators to provide rationales in addition to labels, thus collecting explanations *at the same time* as labels. Explanations for ML model behavior are often created at a different time than the labels. The timing of an explanation is closely related to the explainer role; *when* the explanation is constructed depends on *whom* the explainer is. Using the illustration in Figure 2, we now discuss different explanation timings.

#### 4.2.1 Ante-hoc explanations are created *before* the annotator's mapping

An ante-hoc explainer first generates an explanation, which is then used as input for the annotator model. For example, an ante-hoc explainer model first generates an extractive rationale by identifying rationale sentences in an input text. This extractive rationale then replaces the original input text, which the annotator model (e.g., a classification model) uses to produce an output.

The ante-hoc explanation approach has also been referred to as a pipeline (Wiegreffe et al., 2021), select-then-predict (Chrysostomou and Aletras, 2022), and explain-then-predict (Camburu et al., 2018) setup. The annotator model can be trained separately from the ante-hoc explainer model (Yessenalina et al., 2010; Jain et al., 2020) or the models can be trained jointly (Lei et al., 2016; Bastings et al., 2019). In Figure 2, the top row shows an ante-hoc explainer. Note that ante-hoc explanations do not elucidate the annotator's mapping itself: they only show which input the annotator received to perform the task.

#### 4.2.2 Intra-hoc explanations are created *during* the annotator's mapping

When an explanation is produced *while* the annotator performs the task, such that the roles of explainer and annotator are performed by the same actor, we call this explanation intra-hoc. For example, an ML model annotator may perform a task while “thinking out loud” (Ehsan et al., 2018; Wei et al., 2022), or an ML model may classify a text and provide a free-text explanation at the same time (e.g., Wiegreffe et al., 2021; Wei et al., 2022). We call annotators that are capable of providing rationales while performing a task, thus performing the annotation and explanations tasks simultaneously, *self-rationalizing* annotators.

Alternatively, intra-hoc explanations can be constructed when an ML model is *transparent*. The *transparency* of a model refers to how accessible and interpretable the model's internal mapping from input to output is to humans. Examples of (relatively) transparent ML models include lexicon-based classifiers (Clos et al., 2017) and (small) decision trees.

As described in Section 1, Zaidan et al. (2007) collected human “annotator rationales” by asking human annotators to explain their decisions. Following their terminology, we call explanations that are generated by the annotator itself, *annotator explanations*. Here, the explainer and the annotator are the same actor, so the explainer directly accesses the mapping as performed by the annotator. We therefore consider intra-hoc explanations (e.g., the explanation produced by the annotator in the middle row in Figure 2) to be annotator explanations.

#### 4.2.3 *Post-hoc* explanations are constructed *after* the annotator's mapping

For this type of explanation, the explainer is an external actor that uses the annotator's output to approximate an explanation after the mapping is performed (Ribeiro et al., 2016; Malik et al., 2021). The bottom row in Figure 2 illustrates a *post-hoc* explainer. One advantage of *post-hoc* explainers is that they are usually model-agnostic and applicable to black box models. For example, LIME (Ribeiro et al., 2016), where an ML model learns another ML model's behavior from its outputs, is often used (Carton et al., 2018; Mathew et al., 2021). It is important to keep in mind that *post-hoc* explainers only *approximate* annotator behavior, without actual knowledge about the steps taken inside the annotator model (Jacovi and Goldberg, 2020).

### 4.3 Faithfulness and plausibility

A key question regarding any explanation is whether the explanation is accurate. In XAI, the term *faithfulness* describes whether an explanation accurately reflects the mapping from input to output as performed by the annotator model (Jacovi and Goldberg, 2020). Determining the faithfulness of explanations is challenging, especially for black box models, where the actual mapping from input to output is unknown (Jacovi and Goldberg, 2020; Yin et al., 2022; Lyu et al., 2024). Furthermore, as complete faithfulness may be unattainable, Jacovi and Goldberg (2020) regard faithfulness more as a “greyscale”, rather than as a binary property. The degree to which an explanation is, or can be, (un)faithful will depend on the explanation timing:

- **Ante-hoc:** Ante-hoc explainers do not access the annotator's internal mapping. Instead, the explanation consists of a modified input (e.g. a selection of words) that allows the annotator model to (better) perform the task. Using this modified input, ante-hoc explainers allow the explainee to infer which input features are (ir)relevant for the output (Jain et al., 2020; Chrysostomou and Aletras, 2022). However, the explanations do not explain *how* the annotator generated an output, and may even be barely associated with model outputs (Wiegreffe et al., 2021).
- **Intra-hoc**: A completely faithful explanation can arguably only be constructed when the inner workings of the ML model are known and interpretable, which holds for completely transparent, intra-hoc explainer models (Jacovi and Goldberg, 2020). The faithfulness of intra-hoc explainers like the attention mechanism (Jain and Wallace, 2019; Bibal et al., 2022) and self-rationalizing models (Wiegreffe et al., 2021; Lyu et al., 2023; Turpin et al., 2024) remains unclear and under debate.
- ***Post-hoc***: In contrast to intra-hoc explainers, *post-hoc* explainers do not have access to the annotator's mapping itself. There is therefore no guarantee that *post-hoc* explainers are fully faithful. For example, *post-hoc* explainers that rely on perturbations to approximate the mapping are sensitive to adversarial attacks (Slack et al., 2020). Moreover, *post-hoc* explainers often rely on the Linearity Assumption,^3^ not taking into account that removing parts of the input might unintentionally create out-of-distribution inputs (Hase et al., 2021).

Related to faithfulness is *plausibility*, which describes whether humans find the explanation *convincing* (Jacovi and Goldberg, 2020). In the literature, plausibility is used to describe various notions related to human perception of an explanation, for example, the interpretability (Wood-Doughty et al., 2022), persuasiveness (Herman, 2017), sensibility (Zhong et al., 2019), usefulness (Chiang and Lee, 2022), or the degree to which the explanation is similar to human-annotated explanations (Vafa et al., 2021; El Zini et al., 2022; Schlegel et al., 2022).

### 4.4 Rationales: human-friendly explanations

How an AI system should be explained depends on the explainee and the explanation goal; for example, ML model developers require more technical explanations than domain experts or lay users. For non-technical users, the human-friendliness of an explanation may be much more important than its faithfulness (Carvalho et al., 2019).

Since humans often explain their behavior through natural language (Section 2), rationales can be considered a promising vehicle for conveying explanations about ML models and their behavior in a human-friendly way (Miller et al., 2017). Nonetheless, human-friendliness may be affected by various factors; for example, high-granularity rationales (e.g., words) may require more context to explain in a human-friendly manner, or rationales may be incoherent when the model uses (for humans) illogical heuristics to solve the task. Moreover, humans might prefer using abstractive rationales (e.g., a comment) over extractive rationales (e.g., highlighting words) to explain their decisions. Possible approaches to improving the human-friendliness of rationales may be combining high-granularity rationales like words into sentences (e.g., changing “awesome”, “film” to “this is an awesome film”) (Meldo et al., 2020; Mukhtar et al., 2023), or adding more contextual information justifying the model's behavior.

## 5 Human-annotated rationales in explainable text classification

In this section, we discuss the use of hARs in explainable text classification. In Section 5.1, we first briefly discuss rationales generated by ML models, called model-annotated rationales (mARs). Then, we describe various metrics used to determine agreement between mARs and hARs (Section 5.2). Finally, we discuss how hARs can be used to generate mARs (Section 5.3).

### 5.1 Model-annotated rationales

As discussed in Section 2, mARs are natural language explanations provided by an ML model. Note that mARs can be provided by the annotator (i.e., the classification model), but also by another ML model (i.e., the explainer model). Such a separate explainer model can provide explanations before or after the annotator model maps an input to an output (see Section 4.2). Like hARs, model-annotated rationales (mARs) can be categorized according to the framework we introduced in Section 2.

#### 5.1.1 Form

##### 5.1.1.1 Granularity

Model-annotated rationales come in various granularities: words (e.g., Martens and Provost, 2014; Lundberg and Lee, 2017; Hayati et al., 2021), snippets (e.g., Carton et al., 2018; Sharma et al., 2020; Shen et al., 2022), sentences (e.g., Glockner et al., 2020; Malik et al., 2021), and paragraphs (Chalkidis et al., 2021).

##### 5.1.1.2 Extractive and abstractive

Both extractive and abstractive mARs can be generated; identifying features in the input text that the ML model used to make a classification (e.g., Yessenalina et al., 2010; Ribeiro et al., 2016) results in extractive rationales. Abstractive mARs are created when an ML model generates natural language explanations for its predictions (e.g., Costa et al., 2018; Sap et al., 2020).

##### 5.1.1.3 Categorical and numerical

Most hARs are categorical (see Section 2), e.g., annotators have selected text spans that explain their decision. However, mARs are often numerical values assigned to text spans, like attention weights (Bao et al., 2018; Sen et al., 2020) or saliency maps (Mohseni et al., 2021). Nevertheless, mARs can also be categorical. For example, explainers that first select a subset of the input (Jain et al., 2020), or explainers that perform discrete optimization by applying binary masks on the input (Lei et al., 2016; Bastings et al., 2019).

#### 5.1.2 Exhaustiveness

The exhaustiveness of a mAR is highly dependent on the type of explainer; some explainers aim to produce exhaustive mARs by identifying all text spans that explain the annotator's output, for example, attention, gradient-based, or occlusion-based explainers (Bao et al., 2018; Hayati et al., 2021; Malik et al., 2021). Nevertheless, sometimes a selection is made to limit the number of selected text spans. For example, requiring the rationale size to be less than a fixed percentage of the input text (Lei et al., 2016), choosing a target rationale length (Shen et al., 2022), using a threshold to select high-scoring text spans (Chalkidis et al., 2021; Herrewijnen et al., 2021), or selecting a single most informative sentence (Glockner et al., 2020).

### 5.2 Evaluating model-annotated rationales

Similar to how human-provided labels are often treated as “gold labels”, human-annotated rationales are often treated as “gold rationales” (Section 3.1). Model-annotated rationales are often compared against human-annotated rationales to analyse whether models make predictions based on similar reasons as humans. For example, low agreement can indicate that the model is focusing on spurious correlations (Srivastava et al., 2020; Jørgensen et al., 2022). Comparing against hARs can also provide valuable insights into other aspects. For example, Sen et al. (2020) measure the correlation between the distribution of *all* hAR and mAR words, to study whether models focus on similar categories of words (e.g., adjectives). Nevertheless, care should be taken when using hARs to evaluate mARs and using hARs to represent “human” reasoning (Sen et al., 2021). A study by Jakobsen et al. (2023) found systematic disagreements between demographic groups that were asked to annotate rationales, suggesting that uniform “human” reasoning may not exist.

We now discuss how the agreement between hARs and mARs can be determined. We survey approaches used in the literature (see Table 2), and suggest metrics that might be suited to calculate the agreement between different types of rationales.

**Table 2:** Overview of work that uses hARs to evaluate their generated mARs.

**Related work**	**Extractive**								**Abstractive**		
	**Categorical**					**Numerical**					
Titov and McDonald (2008)	P	R									
Yessenalina et al. (2010)	P	R	F								
McAuley et al. (2012)		P	R								
Tepper et al. (2013)	P	R	F		IOU-F1						
Marshall et al. (2015)	P	R	F								
Lei et al. (2016)	P										
Bao et al. (2018)				C							
Carton et al. (2018)	P	R	F								
Bastings et al. (2019)	P										
Chang et al. (2020)	P	R	F								
Glockner et al. (2020)	P	R	F								
Paranjape et al. (2020)			F		IOU-F1						
Sap et al. (2020)									R	B	WMD
Sen et al. (2020)						PCC	AUC				
Arous et al. (2021)	P	R									
Chalkidis et al. (2021)			F					mRP			
Guerreiro and Martins (2021)			F								
Hayati et al. (2021)						PCC					
Malik et al. (2021)					IOU-F1				R	B	M
Mathew et al. (2021)			F				AUC				
Mohseni et al. (2021)								MAE			
Sharma et al. (2020)			F		IOU-F1						
Jørgensen et al. (2022)							AUC	RBO_EXT_			
Shen et al. (2022)	P	R	F								
Bujel et al. (2023)	P		F								

Abbreviations are as follows (from left to right): For extractive categorical rationales: Precision, Recall, F1-score, Cosine similarity, and Intersection-Over-Union. For extractive numerical rationales: Pearson's Correlation Coeficcient, Mean Absolute Error, Area Under the Precision-Recall curve, mean R-Precision, extrapolated Rank-Biased Overlap. For abstractive rationales: Rouge, Blue, Meteor, and Word Mover's Distance.

#### 5.2.1 Agreement between rationales

When comparing mARs against hARs, some aspects from the above sections are more relevant than others. We now discuss in more detail how agreement between rationales with different forms and degrees of exhaustiveness can be calculated. We focus on ways to measure agreement between hARs and mARs at the instance level (i.e., an individual text). Furthermore, we mainly focus on extractive mARs, as they are most often compared against hARs.

##### 5.2.1.1 Form

The form of the rationale plays a large role in choosing a suitable metric to calculate agreement. Rationales with different *granularities* should not be mixed: word-level rationales will probably not agree with sentence-level rationales, as such rationales have a different bandwidth (Guerreiro and Martins, 2021).

As shown in Table 2, *extractive* rationales are often evaluated using evaluation metrics for classification or regression tasks, and *abstractive* rationales are usually evaluated using metrics from the Natural Language Generation (NLG) field. When evaluating extractive rationales, it can be useful to determine whether the *position* of text spans is relevant. For example, when explaining a negative sentiment label for the sentence “*I had* great *expectations, but this was not a* great *movie*”, the position of *great* matters. In this case, the task can be framed as predicting values for each text span (e.g., token, sentence). In practice, text spans may not match exactly; token-level agreement metrics on human-annotator rationales also often show variability between annotators (see Section 3.2). To allow more flexibility when matching text spans, DeYoung et al. (2020) propose the IOU-F1 metric, which is a more “forgiving” metric to measure overlap between text spans. For example, when two rationales overlap more than 50% (e.g., “a really nice film” and “really nice”), this metric would count this as agreement.

For rationales with *categorical* values (e.g., a text span is part of a rationale or not), agreement is often measured using classification metrics like accuracy, precision, recall, and F1-score (see Table 2). When the values are numerical, agreement has been calculated using metrics like the mean absolute error (MAE) (Mohseni et al., 2021) and Pearson's R (Hayati et al., 2021).

When hARs are categorical, but mARs are *numerical*, the mARs can be converted to categorical values. However, this may cause information loss: for example, when the words “okay” and “fine” received the scores 0.4 and 0.6, they can be converted to 0 and 1 using a threshold, but this will leave out the relevance of the word “okay”. Therefore, we recommend using metrics applicable for comparing numerical to categorical values, like AUC (DeYoung et al., 2020; Sen et al., 2020). Furthermore, when hARs and mARs are both numerical, metrics for measuring the similarity of rankings, like the extrapolated version of the rank-biased overlap (RBO_EXT_) (Webber et al., 2010; Jørgensen et al., 2022) can be used.

Sometimes the position of text spans is of less importance. For example, when the task is to classify whether a review is about a book (i.e., Figure 1), the phrase “*in this book*” may be a sufficient explanation, no matter where, when, or how often the phrase occurs in the input text. Here, different text similarity metrics could be applied to calculate agreement for both extractive and abstractive rationales. For example, to measure the overlap between words or n-grams in a rationale, metrics like the ROUGE, BLUE, or Meteor can be used (Sap et al., 2020; Malik et al., 2021). Furthermore, to measure the semantic similarity between rationales, metrics like BERTScore, BLEURT, and Word Mover's Distance (WMD) (Sap et al., 2020; Clinciu et al., 2021) are applicable.

##### 5.2.1.2 Exhaustiveness

To correctly interpret agreement metrics, it is important to know the exhaustiveness of a rationale. Suppose human annotators were asked to annotate all sentences that support their decision to classify a movie review as positive or negative. In this case, the goal was to collect exhaustive hARs. When such hARs are then compared to mARs that are less exhaustive (e.g., an explainer that only selects the three most important sentences), it cannot be expected that the mARs contain all sentences included in the hARs (i.e. recall is likely to be low). In this case, precision-oriented metrics [e.g., precision or mean R-Precision (mRP)] may be more suitable. Conversely, when human annotators were not asked to annotate all supporting evidence, but the mARs do include all text spans that support a decision, recall-oriented metrics may be more suitable. When both the hARs and mARs are non-exhaustive, agreement in terms of precision and recall is expected to be lower and more difficult to interpret.

Rationales that use different words may still describe similar concepts. For example, in a review topic classification task (i.e. *is this review about a book?*), a human might have highlighted “a well-written novel” as a rationale, while an ML model explainer identified the snippet ‘an engaging book' as a rationale. Then, both rationales are very similar semantically, but use different words. In such cases, evaluation metrics that take into account the semantics of the text (e.g., BERTScore) could be considered to determine agreement between the two rationales.

#### 5.2.2 How should hARs be used in mAR evaluation?

Some work calculates the agreement between multiple human annotators (Carton et al., 2018; Malik et al., 2021), and find that even when human annotators agree on a label, they often do not completely agree when it comes to rationales. If the agreement between hARs is low, we believe it is likely that the agreement between hARs and mARs is also low.

In addition to calculating agreement between hARs and mARs, hARs can be used to put the evaluation scores of mARs into context. For example, mARs are sometimes evaluated by asking users to perform a classification task, replacing the original input with mARs (e.g., Raḿırez et al., 2019; Chang et al., 2020; Jain et al., 2020). However, it might be difficult to interpret the resulting scores without a meaningful baseline. Here, hARs can be used as a reference point for comparing the mAR evaluation scores to. For example, some work replaces the original input text with hARs (Jain et al., 2020; Herrewijnen et al., 2021), finding that humans can accurately make predictions based on human rationales. Where some work evaluates mARs according to their readability (Jain et al., 2020) or length (Shen et al., 2022), it can be informative to compare these scores against evaluation scores for hARs (Jain et al., 2020; Wiegreffe et al., 2021).

Finally, hARs should not be viewed as a benchmark for faithful mARs; Carton et al. (2020) apply faithfulness measures to hARs and ML models, and find that human rationales do not fare well under faithfulness evaluation metrics. This can be expected, as task-solving processes may differ between ML models and humans (Sen et al., 2021; Ju et al., 2022).

### 5.3 Generating model-annotated rationales

Apart from using hARs to *evaluate* mARs, hARs can also serve as examples from which ML models can learn to *generate* mARs. One effect of using hARs to generate mARs, is that the resulting mARs are likely to resemble hARs. A positive aspect of this is that the mARs are more likely to be human-friendly (Section 4.4). A possible downside of this is that the generated mARs might not faithfully represent model behavior, since the mARs are based on human annotator behavior (Section 4.3).

In this section, we will discuss how hARs have been used to train ML models to generate natural language explanations (i.e., rationales). Table 3 provides an overview of work that uses hARs to generate mARs for text classification models.

**Table 3:** An overview of work that uses hARs to generate mARs for text classification tasks.

	**Dataset**	**Form**	**Granularity**	**Value type**	***Post-hoc***	**Ante-hoc**	**Intra-hoc**	**Method**
Tepper et al. (2013)	CPIS/PNA	E	S	C		A		Supervised explainer
Zhang et al. (2016)	IMDB, RoB	E	S	N		A		Supervised explainer
Bao et al. (2018)	BeerAdvocate	E	Sn	N			I	Attention regularization
Strout et al. (2019)	IMDB	E	S	C			I	Supervised explainer
Chang et al. (2020)	IMDB, BeerAdvocate	E	Sn	C		A		Supervised explainer
Glockner et al. (2020)	IMDB	E	Se	C			I	Supervised explainer
Herrewijnen (2020)	IMDB	E	S	C		A		Supervised explainer
Jain et al. (2020)	IMDB	E	Sn	C		A		Supervised explainer
Sap et al. (2020)	SBIC	A	Sn	C			I	NLG model
Arous et al. (2021)	Wiki-tech, Amazon	E	Sn	C	P		I	Attention regularization
Guerreiro and Martins (2021)	IMDB, BeerAdvocate, SST	E	Sn	C			I	Attention regularization
Mathew et al. (2021)	HateExplain	E	Sn	C			I	Attention regularization

Form is abbreviated as Abstractive and Extractive. Granularity is abbreviated as Paragraphs, Sentences, Snippets, and Words. Value type is abbreviated as Categorical and Numerical.

#### 5.3.1 Ante-hoc

An ante-hoc explainer model *first* generates an explanation, which is then used by an annotator model to perform a task (e.g., classification) (Section 4.2). Using hARs, ante-hoc explainer models can learn to construct rationales. For example, Tepper et al. (2013) train an explainer model on hARs to identify rationale sentences in a text, which are then used as input features for their medical classification model. Furthermore, explainer models can learn to generate abstractive mARs from hARs, which can be used as input for the annotator model (Wiegreffe et al., 2021). The latter is comparable to the data generation aim as described in Section 3.1, but with the focus on explainability.

#### 5.3.2 Intra-hoc

In intra-hoc explanation setups, explanations are constructed *while* the annotator model performs the task (Section 4.2). Annotator models can use hARs as “guidelines” for performing the task. For example, using attention regularization, an attention layer (Bibal et al., 2022) is encouraged to focus on the same text spans as the human rationale examples (Bao et al., 2018; Kanchinadam et al., 2020; Pruthi et al., 2022, inter alia). After training, the attention layer can be inspected to identify rationale tokens. Moreover, a *self-rationalizing* annotator model can learn to simultaneously classify a text and generate a rationale based on pairs of human labels and hARs (Sap et al., 2020; Wiegreffe et al., 2021). One example of a self-rationalizing annotator that generates abstractive rationales, is a large language model that is prompted to produce a chain of thought (CoT). Here, the annotator encapsulates the explanation within the output (Wei et al., 2022).

#### 5.3.3 *Post-hoc*

One understudied research direction is using hARs to generate rationales *after* the annotator model has produced an output (i.e., a prediction). One example from the field of text summarization is work by Li et al. (2020), who construct abstractive summaries from keywords in the input texts. When applied to explainable text classification, such strategies could be applicable to constructing low-granularity mARs (e.g., sentences) from high-granularity mARs (e.g., words) and human examples of low-granularity hARs.

## 6 Conclusion and discussion

In this survey, we have given an overview of natural language explanations, also called rationales, in explainable text classification. Throughout this survey, we have focussed on “annotator rationales” as introduced by Zaidan et al. (2007), which are human-annotated highlights explaining “why” a text should receive a particular label. In this section, we provide a concrete list of recommendations for using human-annotated rationales (hARs) in explainable text classification.

### 6.1 Collect human-annotated rationales by default

While collecting hARs increases required annotation time, it is beneficial to data quality, task insight, and data richness (Section 3.2). Therefore, we call for including rationale collection in labeling tasks by default, whenever possible.

However, there are classification tasks for which it is difficult to collect high-quality hARs. In fact, for some tasks, computational methods are used precisely because the task itself is difficult to carry out by humans. An example is the task of authorship attribution (i.e. deciding who wrote a text), where fine-grained distributional differences in character n-grams or function words have shown to be effective (Grieve, 2007). Future work should explore the collection of human-annotated rationales across a wider variety of tasks, to further our understanding of their applicability, benefits, and limitations.

### 6.2 Be specific in instructions for collecting human-annotated rationales

The instructions given to a human annotator affect the form (Section 2.1), exhaustiveness (Section 2.2), and inter-annotator agreement (Section 3.2) of the collected rationales. However, the instructions given to human annotators for annotating rationales vary greatly across surveyed work. Knowing which aspects apply to a set of hARs is imperative for using these hARs in explainable text classification (Section 5, Chiang and Lee, 2022). Moreover, we believe that to collect rationales that are consistent in form and exhaustiveness, it is vital to be precise when instructing human annotators to provide rationales. Work that tailors annotation instructions, aiming to model the human decision-making process (e.g., Lamm et al., 2021; Ray Choudhury et al., 2023), might be an inspiring starting point for constructing precise instructions for rationale collection tasks.

### 6.3 Exploit human-annotated rationales for ML model training

Human-annotated rationales are often collected with the aim of improving ML model training (Section 3.1). Their use during model training has led to improved performance on various classification tasks. Therefore, we believe rationales have great potential for the training of ML models. Furthermore, hARs may prevent models from learning spurious correlations. Whether these benefits hold for other NLP tasks (e.g., Carton et al., 2022), is a question that future research should investigate.

### 6.4 Be cautious with using hARs as “gold rationales”

In Section 5, we looked into the usage of hARs in explainable AI. As described in Section 5.2.1, hARs can be used as gold rationales that mARs should agree with. However, because the hARs and mARs are often not comparable (e.g., in terms of their form and in terms of their exhaustiveness), comparing them can be misleading or uninformative (Section 5.2.2). Therefore, we believe that when comparing two rationales, it is imperative to take into account the (differences in) form and exhaustiveness of these rationales.

Furthermore, when using hARs as gold rationales, it needs to be established why, and for what purposes, these hARs can justifiably be considered to be gold rationales. For example, a specific hAR may be considered an exhaustive reason for a decision, but this may not hold for a hAR collected through different annotation instructions.

Finally, agreement between rationales has been calculated using various approaches in the literature, but a unified approach is lacking. Future work should focus on developing clear and uniform metrics for calculating rationale agreement. Aside from calculating the agreement between hARs and mARs, we recommend using hARs as a baseline for various NLG and explainability evaluation metrics (Section 5.2.2). For example, by comparing the readability score of an mAR to the readability score of a hAR, the mAR's scores can be put into context.

### 6.5 Use hARs as inspiration for generating (human-friendly) mARs

Natural language allows humans to provide explanations that are framed in terms of the knowledge of the explainee (Miller et al., 2017). Accordingly, we believe that rationales are a promising format for explaining ML model behavior in a manner that is human-friendly. Furthermore, because rationales can use domain-specific jargon, we expect that such rationales are an especially suitable explanation format for explaining ML model behavior to domain experts.

In Section 5.3, we briefly discussed how hARs can act as example explanations that explainer models can learn from. One advantage of using hARs as examples for generating mARs, is that the generated mARs are more likely to be human-friendly. Therefore, we believe that hARs form a foundation for explaining the decisions of AI systems to non-technical users working with these systems.

### 6.6 Final remarks and future work

In this survey, we have highlighted the potential of human-annotated rationales for explainable text classification. Some of our recommendations call for further research. For example, we believe that the scientific community would benefit from the construction of new datasets containing human-annotated rationales. Moreover, we believe that it would be important to investigate how, when, and for what tasks, human-annotated rationales can aid data collection and model training. Finally, our findings suggest that human-annotated rationales are not limited to NLP alone, but that they are a promising tool for other areas of research as well, which has the potential to enrich the entire field of XAI.

## Author contributions

EH: Conceptualization, Visualization, Writing—original draft. DN: Conceptualization, Supervision, Writing—review & editing. KD: Supervision, Writing—review & editing. FB: Supervision, Writing—review & editing.

## Footnotes

^1^ https://utrechtuniversity.github.io/annotator-rationales-survey/

^2^We only include rationales provided by the decision-maker in this overview, excluding rationales that are provided by other actors and rationales for pre-defined decisions like in Clinciu et al. (2021). Furthermore, we exclude annotator rationales for tasks outside the scope of this work (see Section 1) (e.g., Srivastava et al., 2017; Camburu et al., 2018; Khashabi et al., 2018; Yang et al., 2018; Atkinson et al., 2019; Meldo et al., 2020; Zhang et al., 2020; Yao et al., 2023). See work by Wiegreffe and Marasovic (2021) for an overview of datasets for explainable NLP.

^3^The assumption that different parts of the input independently influence an annotator's output (Jacovi and Goldberg, 2021).
